# Immunomodulatory actions of methotrexate on T cells in juvenile idiopathic arthritis

**DOI:** 10.1186/1479-5876-10-S3-P40

**Published:** 2012-11-28

**Authors:** Maja Bulatović Ćalasan, Sebastiaan Vastert, Frederik Verweij, Nico Wulffraat, Femke van Wijk, Berent Prakken

**Affiliations:** 1 Dept. of Pediatric Immunology, University Medical Centre Utrecht, Wilhelmina Children’s Hospital, Utrecht, the Netherlands

## Introduction

In the pathophysiology of juvenile idiopathic arthritis (JIA), T cells play an important role. The balance between immune activation, governed by effector T cells (Teff) and immune regulation, governed by regulatory T cells (Treg), is disturbed. Low-dose methotrexate (MTX) is the most commonly used drug to induce disease remission in JIA. Nevertheless, despite its use for decades as the prime anti-inflammatory drug, its immunomodulatory actions *in vivo* remain poorly elucidated. We hypothesized that MTX restores the immune balance by increasing Treg number and suppressive function and decreasing Teff activation status and effector functions.

## Aim

To determine the immunomodulatory effects of MTX on Treg and Teff in JIA during MTX treatment.

## Methods

Peripheral blood mononuclear cells (PBMCs) of JIA patients were isolated before MTX start (T0), at 3 (T3) and 6 months (T6) during MTX treatment. Frequency and phenotype of CD4+FoxP3+ Treg were analyzed *ex vivo* by flow cytometry, and their suppressive function was analyzed in CFSE suppression assays. Proliferation of CD4+ and CD8+ Teff was determined with CFSE, upon a 5-day culture in the presence of a-CD3. Cytokine production was measured *ex vivo* upon short PMA/ionomycin stimulation by flow cytometry as well as in 5-day culture supernatants and in plasma by luminex.

## Results

Treg frequency and suppressive capacity did not increase during MTX treatment. Frequency of naïve CD4+CD45RA+ Teff increased, whereas the memory CD45RO+ Teff decreased at T3 vs. T0. CD4+ and CD8+ Teff proliferation was significantly increased at T6 (mean: 75.3 +/- SD 14.9) vs. T0 (mean: 58.5 +/- SD 17.9) (p=0.002) in patients with good and poor MTX response. *Ex vivo* and supernatant cytokines showed no differences between time-points, however plasma IFNγ was markedly increased at T6 vs. T3 and T0.

## Conclusion

We observed no effects of MTX on Treg. In contrast to our hypothesis, Teff increased their proliferation during 6 months of MTX treatment, which was accompanied by increased plasma IFNγ. Taken together, this suggests immunomodulatory rather than immunosuppressive effects of low-dose MTX treatment in JIA. We are now investigating the role of antigen-presenting cells in increased T cell proliferation during MTX treatment.

